# Case of left ventricular thrombus managed with thrombectomy with left ventricular reconstruction in a patient who had coronavirus disease 2019 infection

**DOI:** 10.1186/s13019-023-02108-5

**Published:** 2023-01-07

**Authors:** Kenji Suzuki, Shun-Ichiro Sakamoto, Atsushi Hiromoto, Yusuke Motoji, Ryosuke Amitani, Takako Yamaguchi, Yosuke Ishii

**Affiliations:** 1 Department of Cardiovascular Surgery, Nippon Medical School Musashikosugi Hospital, 1-383 Kosugimachi, Nakahara-Ku, Kawasaki, Kanagawa 211-8533 Japan; 2 Department of Nursing, Nippon Medical School Musashikosugi Hospital, 1-383 Kosugimach, Nakahara-ku, Kawasaki, Kanagawa 211-8533 Japan; 3 Department of Cardiovascular Surgery, Nippon Medical School, 1-1-5 Sendagi, Bunkyo-ku, Tokyo, 113-8602 Japan

**Keywords:** Left ventricular thrombus, COVID-19, Left ventricular reconstruction, Bovine pericardial patch

## Abstract

**Background:**

Intracardiac thrombus is relatively rare in patients with coronavirus disease 2019 (COVID-19). However, if it occurs, thrombotic complications are likely to develop. In this case, we performed a successful thrombectomy on a patient who developed left ventricular thrombus after COVID-19 infection without complications.

**Case presentation:**

A 52-year-old man sought medical care due to fever, dyspnea, and abnormalities in the taste and smell that persisted for 2 weeks. The patient was diagnosed with COVID-19 and was treated with remdesivir, baricitinib, and heparin. Three weeks after hospitalization, electrocardiogram revealed angina pectoris, and cardiac catherization showed left anterior descending coronary artery stenosis. In addition, global hypokinesis and a thrombus at the left ventricular apex were observed on echocardiography. Left ventricular reconstruction concomitant with coronary artery bypass grafting was performed. A thrombus in the left ventricle was resected via left apical ventriculotomy, and the bovine pericardium was covered and sutured on the infarction site to exclude it. The patient was extubated a day after surgery and was transferred to another hospital for recuperation after 20 days. He did not present with complications.

**Conclusions:**

Thrombotic events could be prevented via thrombectomy with left ventricular reconstruction using an intraventricular patch to exclude the residual thrombus.

## Background

Intracardiac thrombus is relatively rare in patients with coronavirus disease 2019 (COVID-19), and it is identified via pathological autopsy in 2.5% of cases [1]. The condition is commonly managed with thrombolytic therapy. However, some patients develop cerebral infarction during the disease course [2–4]. In such cases, it is challenging to confirm whether surgical thrombectomy can be performed. Herein, we report a successful thrombectomy with left ventricular (LV) reconstruction in a patient who developed LV thrombus after COVID-19 infection.

Written informed consent for publication of case details and images was obtained from the patient before drafting of the manuscript.

## Case presentation

A 52-year-old man with autism did not immediately seek medical care despite having fever, dyspnea, and abnormalities in the taste and smell. Emergency assistance was sought as the symptoms persisted for 2 weeks. The patient was not vaccinated against COVID-19. His body temperature upon admission was 37.5 °C; pulse rate, 124 beats/min; respiratory rate, 16 cycles/min; blood pressure, 145/106 mmHg; and oxygen saturation on room air, 93%. The blood biochemical findings were as follows: white blood cell count, 6730/µL; platelet count, 282,000/µL, C-reactive protein level, 8.73 mg/dL; creatinine level, 1.06 mg/dL; prothrombin time-international normalized ratio, 1.11; activated partial thromboplastin time, 33.4 s; fibrinogen level, 625.2 mg/dL; and D-dimer level, 3.43 µg/mL. Based on the polymerase chain reaction test for COVID-19, the cycle threshold value was 22.3, and chest radiography revealed pneumonia. The patient was diagnosed with COVID-19 and was treated with remdesivir, baricitinib, and heparin. However, steroids were not administered. The respiratory symptoms improved, and the patient was transferred to another hospital for recuperation 3 weeks after hospitalization.

Electrocardiogram at the time of transfer revealed ST elevation with QS pattern in V2–V5, and cardiac catherization showed #6 99% stenosis and #7 chronic total occlusion (with collateral circulation from the right coronary artery). In addition, global hypokinesis and a thrombus (34 × 18 mm) at the LV apex were observed on echocardiography. The patient was re-transferred to our hospital for surgery 4 days after the first transfer.

**Fig. 1:**
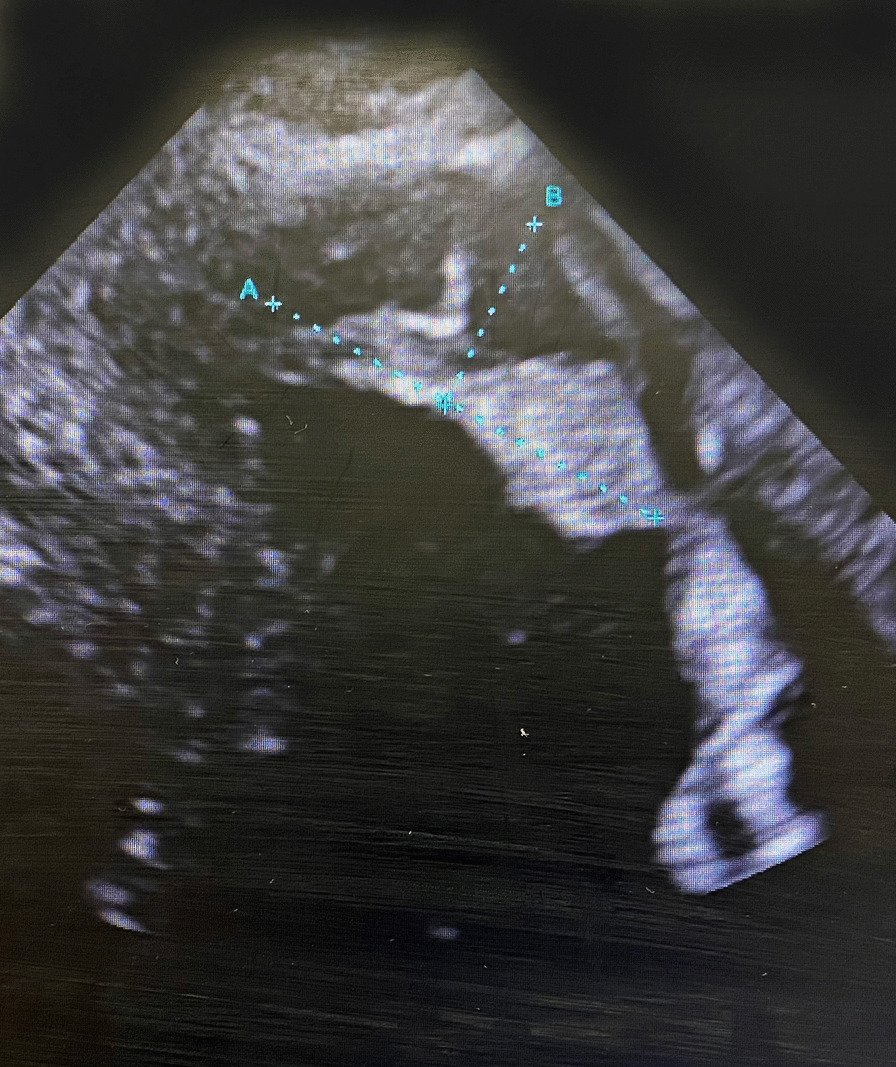
Preoperative echocardiography revealed a partially mobile thrombus at the LV apex

During re-transfer, the blood biochemical findings were as follows: white blood cell count, 5340/µL; platelet count, 329,000/µL; C-reactive protein level, 3.79 mg/dL; N-terminal pro-brain natriuretic peptide level, 1167 pg/mL; creatinine level, 0.82 mg/dL; prothrombin time-international normalized ratio, 1.07; activated partial thromboplastin time, 42.6 s; fibrinogen level, 605.5 mg/dL; D-dimer level, 2.45 µg/mL; ATIII, 80.6%; protein C antigen, 120%; and protein S antigen, 135%. Chest radiography revealed a cardiothoracic ratio of 50% and the absence of pneumonia and pulmonary congestion. Electrocardiogram showed the following: sinus rhythm, pulse rate of 85 beats/min, negative T-waves in V1–V5, and QS pattern in V1–V4. Echocardiography showed an LV ejection fraction of 51%, LV hypokinesis, and a partially mobile thrombus (26 × 13 mm) at the LV apex (Fig. 1). Head computed tomography revealed no abnormal findings such as cerebral infarction.

**Fig. 2:**
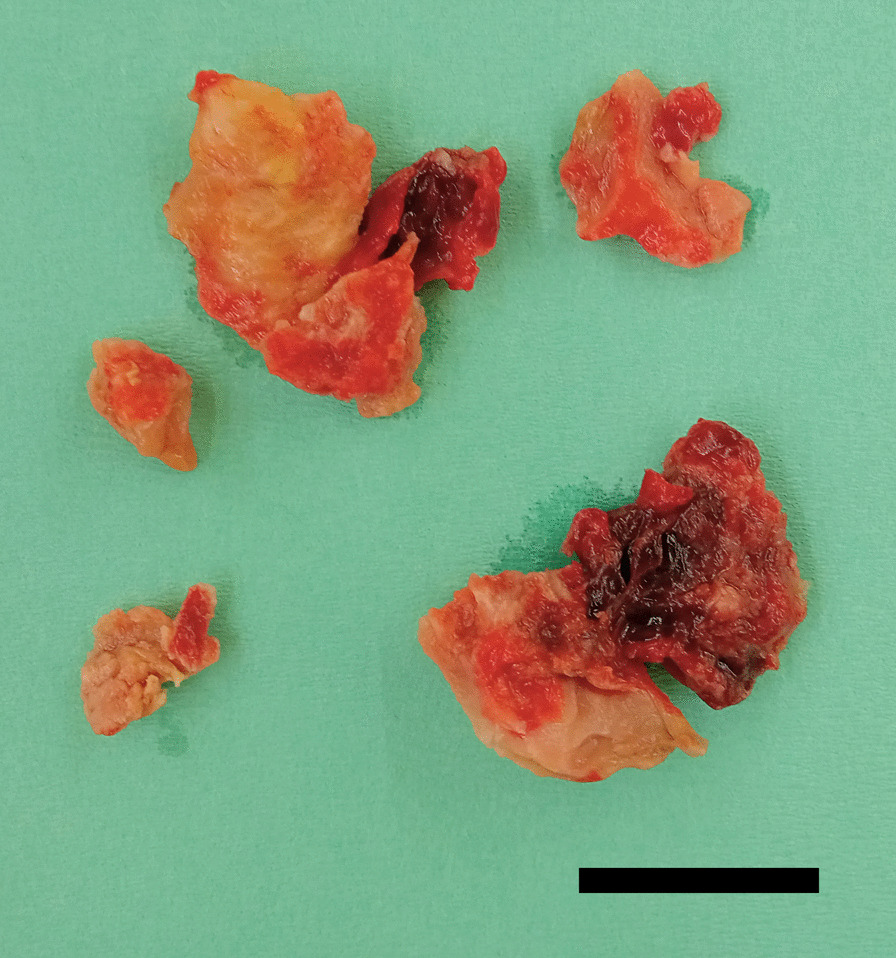
The excised thrombus (bar = 20 mm)

**Fig. 3:**
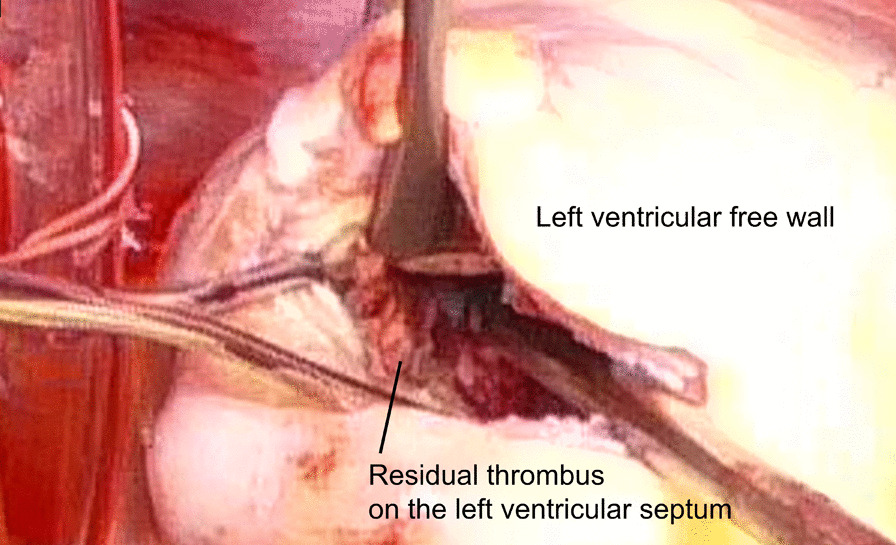
The residual thrombus adhered to the LV septum

**Fig. 4:**
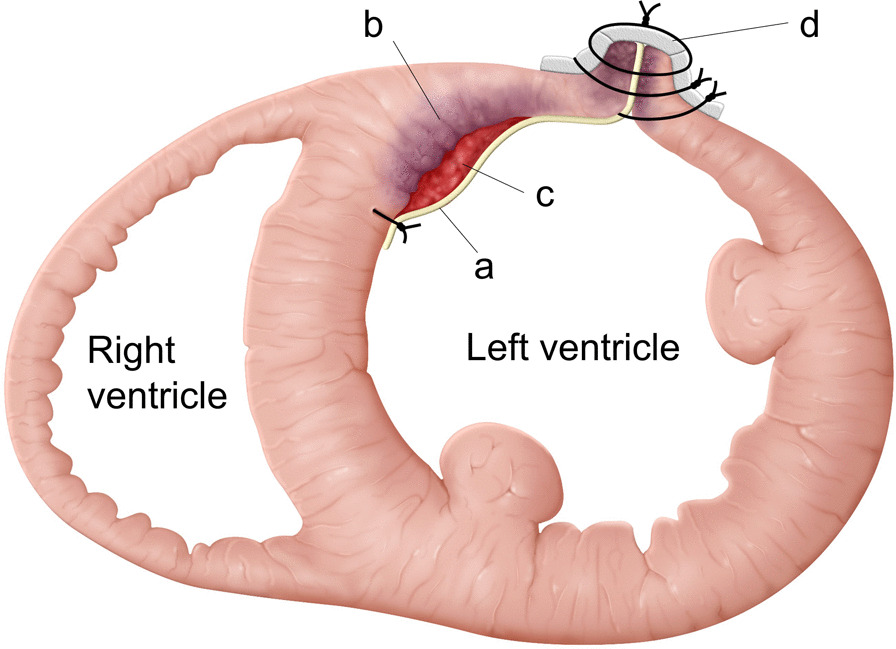
The thrombus was resected via apical ventriculotomy. The bovine pericardium (**a**) was covered and sutured on the infarction site (**b**) to exclude the residual thrombus (**c**) attached to the left ventricular wall. The left ventricle was sutured using felt strips (**d**)

LV reconstruction concomitant with coronary artery bypass grafting was performed on day 4 after re-transfer. After aortic cross clamping, an organized thrombus (Fig. 2) was resected via apical ventriculotomy. The thrombus was connected to the septal and free wall (Fig. 3) with visible disruption of the endocardium due to myocardial infarction. Therefore, the bovine pericardium was covered and sutured on the infarction site to exclude it (Fig. 4). The left ventricle was sutured using felt strips; then, left internal thoracic artery–left anterior descending grafting was performed.

The patient was extubated a day after surgery. Drainage was required for pericardial effusion. However, there were no other complications. Postoperative echocardiography revealed the absence of residual LV thrombus. The patient was prescribed with oral aspirin, warfarin, and diuretic and was transferred to another hospital for recuperation 20 days after the surgery. The thrombus collected during surgery was examined histopathologically and was found to be an organized thrombus with inflammatory cells in some parts.

## Discussion and conclusions

To the best of our knowledge, this is the first case report about a successful thrombectomy with LV reconstruction in a patient who developed LV thrombus after COVID-19 infection. Janula et al. managed a patient with the same condition. However, thrombectomy using the transaortic valve approach with aortotomy was performed [5].

The patient herein did not exhibit clinical factors of LV thrombus after myocardial infraction. The risks of LV thrombus following myocardial infarction include large infarct size, severe apical asynergy, LV aneurysm, and anterior myocardial infraction [6]. Moreover, it is reported that approximately 80% LV thrombus adhere to the apex of the heart [7]. In this case, however, although the patient had anterior myocardial infarction, the wall motion was mildly depressed, and the thrombus was connected to the septal and free wall.

The causes of thrombus formation in patients with COVID-19 are enhanced blood coagulation system, suppressed fibrinolytic system, and damaged cardiovascular endothelium. D-dimer, which is the product of fibrin degradation after thrombus degradation by the fibrinolytic system, is a useful index for determining the presence of thrombus [8]. The serum D-dimer concentration of patients with severe COVID-19 is significantly higher than that of patients with non-severe infection [9, 10]. In this case, it was also higher than that during admission for COVID-19 pneumonia. Hence, the patient might be at risk of thrombus formation.

Surgical thrombectomy may be better than pharmacological treatment for LV thrombus [11]. The condition is classified into three types based on its morphology: the mural type, which is firmly attached to the LV wall in the form of a disk and is not mobile; the protruding type, which protrudes into the left ventricle; and the mobile type, which moves independent of the LV wall [12, 13]. In our case, the thrombus is mobile, which is believed to have the highest risk among all types of LV thrombus.

In addition to thrombectomy, we performed exclusion of the LV septum and free wall using a bovine pericardial patch. The design of the LV plasty is similar to that in the David–Komeda procedure in terms of myocardial infarction site exclusion. Minami et al. used the David–Komeda procedure for LV thrombus after acute myocardial infarction to prevent recurrence of thrombus formation [13]. Exclusion aims to cover myocardial tissue disruption with the residual thrombus. In the current case, it was mainly observed in the LV septum, which was assumed to be the origin of thrombus formation toward the free wall. Our case involved recent myocardial infarction, and the site of left ventriculotomy was acceptable for direct suture. Therefore, the bovine pericardial patch is not commonly used to cover the disrupted septum and is doubly fixed with ventriculotomy closure.

We performed thrombectomy with LV reconstruction on a patient who developed LV thrombus after COVID-19 infection. Postoperative thrombotic events could be prevented via patch reconstruction with consideration of the wall thrombus.

## Data Availability

The datasets supporting the conclusions of this article are included in the article.

## Abbreviation

LV: Left ventricle, left ventricular
